# Evaluation of the dual IFNγ/IL-2 fluorospot-assay with flow cytometry for detection of HLA-restricted HIV-specific T-cell responses in HIV controllers

**DOI:** 10.1186/1742-4690-9-S2-P243

**Published:** 2012-09-13

**Authors:** N Keane, C Almeida, S Roberts, I Ahmad, S Mallal, M John

**Affiliations:** 1 Murdoch University, Perth, Australia

## Background

The IFNγ ELISpot assay is used widely for high through-put screening of HIV-specific responses in studies of HIV infection and vaccine studies. However, dual production of IFNγ/IL-2 and increased proliferative capacity may be associated with better natural control of HIV infection. We evaluated a novel fluorospot assay enabling the identification of dual IFNγ/IL-2 producing antigen-specific cells and compared it with intracellular cytokine staining by standard flow cytometry in individuals with natural control of HIV-infection.

## Methods

PBMC from five untreated HIV-infected individuals were stimulated overnight with HIV peptides or controls in dual IFNγ/IL-2 pre-coated plates. Peptide arrays were specifically selected based on the individual HLA type. Secreted IFNγ and IL-2 were detected using fluorescent-conjugated antibodies. Fluorescent spots were enumerated on the iSpot AID reader. Responses were considered positive if >50 spots/million cells SFU after background subtraction.Positive responses were then evaluated by flow cytometry using the Gallios flow cytometer.

## Results

Dual IL-2/IFNγ producing cells were detected to anti-CD3-stimulated PBMC from all patients. IFNγ responses alone were detected to 35 of 136 HLA-restricted peptides tested (median =73, 52-4190 SFU) across the 5 patients (1/19, 5/19, 7/28, 9/15 and 13/55 for each patient), while IL-2 responses were either low grade or undetectable for the majority of HIV peptides tested. Dual IFNγ/IL-2 producing HIV-specific T cells were not detected using the fluorospot assay. 24/35 peptides induced CD8 T cell-IFNγ production by flow cytometry.

## Conclusion

HIV-specific mono-IFNγ responses were detected using the novel fluorospot assay. However limited HIV-specific dual IFNγ/IL-2 responses were detected in this patient group. A greater number of epitope-specific positive responses were detected in the fluorospot compared with flow cytometry suggesting the fluorospot may be more sensitive in detecting a greater breadth of epitope-specific T cell responses, and therefore better for screening purposes than flow cytometric methods.

